# Factors Influencing the Survival Rate of Teeth and Implants in Patients after Tumor Therapy to the Head and Neck Region—Part 1: Tooth Survival

**DOI:** 10.3390/jcm11206222

**Published:** 2022-10-21

**Authors:** Ramona Schweyen, Waldemar Reich, Dirk Vordermark, Thomas Kuhnt, Andreas Wienke, Jeremias Hey

**Affiliations:** 1 Department of Prosthetic Dentistry, University School of Dental Medicine, Martin Luther University Halle-Wittenberg, Magdeburger Str. 16, 06112 Halle, Germany; 2 Department of Oral and Maxillofacial Plastic Surgery, Martin Luther University Halle-Wittenberg, Ernst-Grube-Str. 40, 06120 Halle, Germany; 3 Department of Radiotherapy, University Clinic, Martin Luther University Halle-Wittenberg, Ernst-Grube-Str. 40, 06120 Halle, Germany; 4 Department of Radiotherapy, University Clinic, University Leipzig, Stephanstr. 9a, 04103 Leipzig, Germany; 5 Department of Medical Epidemiology, Biometry and Computer Science, Martin Luther University Halle-Wittenberg, Magdeburger Str. 8, 06112 Halle, Germany

**Keywords:** head and neck cancer, tooth loss, risk factors, radiation therapy

## Abstract

We aimed to evaluate possible factors influencing the long-term survival of teeth after tumor therapy (TT) to the head and neck region with and without radiation. Between January 2019 and January 2020, patients who underwent TT for head and neck cancer and received dental treatment before and after TT at the Department of Prosthetic Dentistry of the Martin Luther University Halle-Wittenberg were enrolled in the study. Clinical examination with assessment of dental status and stimulated salivary flow rate (SFR) was performed and information about disease progression and therapy was retrieved from medical records. Of 118 patients (male: 70.3%; mean age: 63.2 ± 12.4 years), 95 received radiotherapy (RT), and 47 were administered radio-chemotherapy (RCT). The teeth of irradiated patients exhibited a lower 5-year survival probability (74.2%) than those of non-irradiated patients (89.4%). The risk of loss (RL) after RT increased with nicotine use, presence of intraoral defects, reduced SFR, RCT and regarding mandibular teeth, and decreased with crowning following TT. Lower SFR increased the RL even without RT. Consideration of patient’s treatment history, individual risk profile, and clinical findings during the prosthetic planning phase could enable earlier, more targeted dental treatment after TT (e.g., timely crowning).

## 1. Introduction

After tumor therapy (TT) in the head and neck region, the treating dentist is responsible for follow-up care, and often, extensive dental-prosthetic rehabilitation is required; moreover, the side effect profiles of patients differ considerably. In addition to resection-related anatomical changes, restrictions in mouth opening, and tongue and mandibular mobility, patients who undergo additional radiation as part of TT often experience pronounced dry mouth [1,2,3]. The resulting impaired oral hygiene and increased susceptibility to caries often lead to the development of radiation caries, for which a multifactorial pathogenesis is discussed [4,5,6]. In several cases, cooperation between the dentist and patient racing against tooth decay begins after TT. Therefore, it is often necessary to focus on the preservation of a few teeth which are strategically important from a prosthetic perspective, such as the canines and premolars. After removal of the other teeth, they can usually be used to anchor removable dentures and remain readily accessible to cleaning even with limited mouth opening [4,7]. After extensive filling therapy, the dentist usually advises crowning of the remaining teeth, particularly those with high prosthetic value. In this case, the crown margin is usually positioned as subgingivally as possible in order to prevent crown margin or root caries. In everyday clinical practice, however, radiation caries also progresses on crowned teeth in the cervical region after TT and, despite all efforts, can make it necessary to remove the teeth and rework or remake the denture within a very short time [4,7].

Currently, various factors, such as sex, age, allocation of teeth to the jaw, type of restoration of previously damaged dental hard tissue (e.g., crowning), or stimulant abuse (e.g., nicotine) are believed to influence the survival of original teeth even in patients without a tumor history [8,9,10]. In patients after TT to the head and neck region, various other aggravating factors are added, such as the side effects of radiotherapy (RT), possibly in combination with chemotherapy (RCT), tumor location, and the presence of intraoral defects, which make accessibility to the oral cavity more difficult for both the practitioner and the patient for home oral hygiene [2,4]. In particular, RT is considered of substantial value regarding the prognostic evaluation of the remaining teeth. Therefore, despite developments in irradiation techniques, removal of questionable or critical teeth before RT is considered absolutely essential to prevent subsequent surgical removal of the destroyed teeth with the risk of developing dentogenic osteoradionecrosis [11,12]. Clinically, however, it often appears that patients who were not irradiated during the course of TT are also exposed to an increased risk for progressive tooth decay with a similar need for rehabilitation. To date, no studies are available that systematically evaluate the various possible factors influencing tooth survival after TT in the head and neck region.

The present study aimed to evaluate the dental extraction requirements of patients before and the survival of teeth after TT in the head and neck region. It was postulated that there is no relevant difference between irradiated and non-irradiated patients. In addition to irradiation, the influence of factors such as age, sex, assignment of teeth to the jaw, nicotine abuse, stimulated salivary flow rate (SFR), chemotherapy, crowning of teeth before or after TT, and the presence of intraoral defects on the survival probability of teeth after TT was also evaluated.

## 2. Materials and Methods

One hundred fifty-three patients who underwent surgical resection and/or RT/RCT due to tumor disease in the head and neck region between 1985–2018, and were undergoing dental follow-up after completion of TT during 1 January 2019, to 31 January 2020, at the University Clinic for Prosthodontics were included in the present study. Thereof, 35 patients with advanced age- or disease-related general health or cognitive impairment, premature death and not provided informed consent were excluded from the study. The conduct of the study was approved by the Ethics Committee of the Medical Faculty of Martin Luther University Halle-Wittenberg (Nos. 2017-62 and 2018-130).

### 2.1. Tumor Therapy

The type of TT was determined and conducted by the treating clinics, mostly oral and maxillofacial surgery, otorhinolaryngology, and radiotherapy at the University Hospital Halle. In the presence of appropriate histopathological findings, operability of the patient, and consent of the same, surgical removal of the tumor was performed. The teeth in direct relation to the tumor or the area of the safety distance as well as the teeth that could not be preserved because of general decay were mostly removed during tumor removal surgery. If primary coverage was not possible, defect closure via local or microvascular anastomosed flaps was advised. If primary or adjuvant RT was required, it was individually determined with regard to the applied radiation technique, dose, and regimen by the specialists and medical physicists of the University Hospital for Radiotherapy. To ensure immobility during radiation sessions, individual head-neck-shoulder masks were fabricated for each patient. In some patients, chemotherapy was administered simultaneously with irradiation. In most cases, cisplatin, if necessary, in combination with 5-fluorouracil, or alternatively carboplatin, was used as short infusions in the 1st and 5th weeks of treatment.

### 2.2. Dental Treatment in Relation to RT

Before RT, all patients were referred to the Department of Dentistry, Oral and Maxillofacial Surgery by the University Clinic for Radiotherapy. The focal dental treatment performed here was based on the recommendations of the German Society for Dental, Oral and Maxillofacial Medicine [13]. Treatment planning was performed by collaboration between an experienced dental or medical staff member of the Department of Prosthodontics and Oral and Maxillofacial Surgery. The aim was to remove teeth with questionable and infamous prognosis (avital and insufficiently root canal-treated teeth, painful teeth potentially with radiographic apical translucency, pronounced pulpal carious lesions, large fillings, fractures or advanced abrasions, probing depths ≥5 mm, furcation involvement, and retained or impacted teeth) at least one week before the start of irradiation [14]. In addition, all teeth were extracted that were not accessible to proper hygiene due to reduced mouth opening. The concept of shortened dentition was aimed at these cases [15]. If this was not feasible, the goal was to preserve the canines for subsequent prosthetic restoration. All dentate patients received a 5 mm-thick polyethylene radiation protection splint. In addition, patients were instructed to load the splints with fluoride-containing gel and to wear them for 5–10 min once per day after brushing their teeth [16]. Finally, patients were informed about the need for meticulous oral hygiene. Patients with removable dentures were advised not to wear them as often as possible to avoid mechanical injury to the vulnerable mucous membranes. Instructions on the handling of the radiation protection splints, oral hygiene, and nutrition during and after RT were provided to the patients in the form of brochures [17,18].

During RT, the oral cavity was examined weekly at the University Clinic for Radiotherapy; patients were reminded regarding oral hygiene if necessary, and a dexpanthenol-containing rinse was prescribed if the degree of mucositis was ≥1. Dental interventions were performed only when necessary.

### 2.3. Dental Treatment after TT

All patients were recommended to follow a frequent quarterly follow-up regimen. This included a dental check-up, professional tooth cleaning, and timely treatment of any defects in the dental hard tissues. In general, only temporary dentures were fabricated or existing dentures were adapted in the first year following TT. The reasons for this were to reduce the risk of treatment failure due to recurrence and to assess the prognosis of the remaining teeth more reliably. In the case of fully edentulous patients or patients adequately provided with fixed dentures, the aim was to maintain the existing tooth status. In the case of residual dentition, the aim was to preserve the teeth with a comparatively good prognosis and use them to anchor removable dentures. In the case of carious lesions, teeth were treated following the recommendations of Kielbassa et al. [19] and Grötz et al. [20]. In the case of progressive generalized decay due to caries, the aim was to crown the remaining teeth as soon as possible.

### 2.4. Data Collection

The following data and findings were obtained from the disease documentation or by clinical examination by one and the same examiner.

Data from medical history and disease documentation:

- Date of birth and sex.
- Tumor disease (tumor location and tumor entity, classification according to the TNM (tumor, node, and metastasis) and UICC (International Union Against Cancer) stages.
  ○ TT (surgical resection [International Classification of Diseases 10th revision (ICD-10) classification, bone surgery, and reconstructive procedures], RT [adjuvant or curative, radiation period, technique, and applied radiation dose], and RCT).
- Date of end of primary TT.
- Tooth removal in relation to TT.
- Information on tooth survival after TT.
  ○ Date of tooth removal.
  ○ Causes of tooth removal (periodontal causes, osteoradionecrosis (ORN), and [radiation] caries [dichotomous classification into “radiation caries” and “no radiation caries”, following the classification of Grötz et al., Grades 2–4 were defined as radiation caries [5,6,20]).
- Stimulant use (nicotine and alcohol).
- Patient’s death during the study period.

Clinical examination:

- Dental status.
- Presence of intraoral defects (defects were defined as areas resected during tumor resection and reconstructed by flaps or grafts, if necessary. The classification was based on that of Nicoletti et al. [21] into:
  ○ No oral resection defects.
  ○ Lateral defect [lateral floor of the mouth, mandible, and cheek pouch, possibly with tongue adhesion].
  ○ Anterior defect [anterior midline crossing the floor of the mouth, intercanine section mandible, and labial vestibule, with tongue adhesion if necessary].
  ○ Central defect [(hemi-)glossectomy, with pronounced tongue adhesion].
  ○ Retromolar defect [retromolar triangle incl. soft palate and tonsil].
  ○ Extensive defects [hemifacial floor of the mouth or mandible from anterior to retromolar, retromolar triangle, soft palate, and tongue margin].
  ○ Hard/soft palate defects [not reconstructed].
  ○ Hard/soft palate defects [reconstructed]).
- Determination of the stimulated salivary flow rate (SFR; saliva samples were collected at least 1 h after a meal in the morning [9:00–11:00 a.m.]). Patients were asked to rinse their mouth and swallow any residual saliva. Afterward, they were instructed to chew on a paraffin pellet [Ivoclar Vivadent; Ellwangen, Germany] for 5 min and to spit the saliva into cups during this time. Therefore, the SFR was calculated (mL/min) [22,23]. According to the classification of Dawes et al. [23], patients’ SFR was finally classified as “high SFR” [>3.5 mL/min], “regular SFR” [1.0–3.5 mL/min], “low SFR” [0.5–1.0 mL/min] and “lowest SFR” [<0.5 mL/min]).

### 2.5. Statistical Analysis

Statistical analysis was performed with the support of the Institute of Epidemiology at Martin Luther University Halle-Wittenberg. First, the epidemiological characteristics of the study cohort were presented descriptively. Data analysis of the survival time of the teeth after TT and evaluation of possible influencing factors were performed using the Kaplan–Meier method and log-rank test. The performance of RT was found to be a significant influencing factor on the survival rate of teeth. Therefore, for the subsequent multivariable analysis, a subdivision was made into “teeth of irradiated patients” and “teeth of non-irradiated patients.” Subsequently, the influence of the variables age, sex, chemotherapy, SFR, assignment of teeth to the jaw, defect situation, tumor location, and crowning on tooth survival was determined. These analyses were performed considering the affiliation of multiple teeth to a patient (clustering) using the marginal model with calculation of hazard ratios and robust 95% confidence intervals [24]. In the marginal model, the inclusion of clustering leads to an increase in standard errors compared with Cox regression. To improve the sharpness of the test, the variables “defect situation” was dichotomized, i.e., divided into “intraoral defect present” and “no intraoral defects”. The time period for the dental survival analyses extended from the end date of the TT to the examination date. Microsoft Excel 2016 (Microsoft Deutschland GmbH, Munich, Germany) and IBM SPSS Statistics, version 25.0 (IBM Inc., Ehningen, Germany) were used for analysis.

## 3. Results

### 3.1. Characterization of the Study Cohort

Initially, data from 118 patients were included in the study (male: 70.3%; age of males: 63.2 ± 12.4 years, age of females: 63.0 ± 13.4 years). Regular tobacco use was reported by 49.4% of men and 37.1% of women. Clinical examination of the patients was conducted at an average of 80 ± 67 months after the completion of primary TT. All patients had undergone dental rehabilitation at this time such that no dental procedures were scheduled in a timely manner.

The majority of patients (80.1%) required TT because of squamous cell carcinomas; the remainder suffered from mucoepidermoid (1.7%), acinar cell (2.5%), lymphoepithelial (1.7%), and adenoid cystic carcinomas (1.7%), as well as sarcomas (2.5%), cervical cancer of unknown primary (CUP) syndrome (2.5%), or other malignancies (6.8%; Table 1).

RT was performed in 95 patients (80.5%). Eleven patients received primary curative irradiation and eighty-four received adjuvant irradiation. The irradiation dose was 64.6 ± 6.1 Gy at initial irradiation. Step-and-shoot intensity-modulated RT (IMRT) was used in 47 (50.5%) patients, volumetric modulated arc therapy (VMAT) in 20 (21.1%), three-dimensional conformal radiation therapy in 17 (17.9%), and 2D-RT in 8 (8.4%) patients. In three patients, the irradiation technique could not be accurately reconstructed. Additional chemotherapy was used in 47 patients (39.8%), mainly for advanced UICC stages III and IV tumors.

### 3.2. Dental Treatment during TT

All patients in whom head and neck irradiation was intended presented to the Department of Dentistry and Oral and Maxillofacial Surgery before initiation and underwent basic dental reconstruction. Teeth that could not be or were not worthy of preservation and those locally related to the malignancy were also removed in patients without subsequent radiation. Of the 118 patients, 19 were already edentulous at the onset of TT. Of the 99 dentate patients, no extractions related to TT were performed in 29 cases. In the remaining 70 patients, a total of 478 teeth were removed (Table 2). On average, four teeth were removed in connection with TT in both dentate patients to be irradiated and those who were not to be irradiated.

In 17 patients (14.4%), 38.5 ± 39 (range: 3–136) months after the end of TT, recurrence was detected. This led to a new resection of the affected tissues in 12 patients, and a second irradiation in five patients. Of these five patients, three required a third radiation treatment due to a second recurrence, and one patient received combined RCT. Eleven patients (11.6%) had been diagnosed with ORN at the time of the study, which required resection of the affected jaw sections. All patients affected by ORN were male and had received primary or adjuvant RT for squamous cell carcinoma of the oral cavity or oropharynx.

### 3.3. Long-Term Survival of Teeth after TT

After TT, 87 patients had a total of 1262 teeth of which 164 were crowned. Two hundred and twelve teeth belonged to 23 patients who had not been irradiated. After TT, radiation caries was diagnosed in 50 patients and 213 additional teeth were crowned. At the time of the study, a total of 343 teeth (27.2%) had to be removed. Indications for tooth extraction were as follows: tooth structure defects (75.5%), hyperplasia after RT in childhood (10.5%), occurrence of ORN (7.6%), periodontal conditions (5.2%), and apical periodontitis (1.2%).

**Figure 1:**
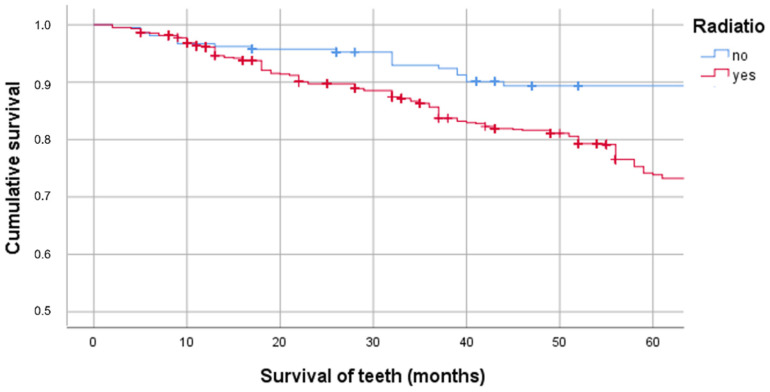
Cumulative survival of teeth in irradiated and non-irradiated patients.

Patients who underwent RT lost teeth more frequently than non-irradiated patients (5-year survival probability [5-YSP] of the teeth of non-irradiated patients, 89.4% [95% confidence interval (CI): 84.8–94.9%] vs. 74.2% [95% CI: 71.0–77.4%] of the teeth of irradiated patients [*p* < 0.001]; Figure 1).

#### 3.3.1. Factors Influencing the Survival of Teeth of Irradiated Patients

**Figure 2:**
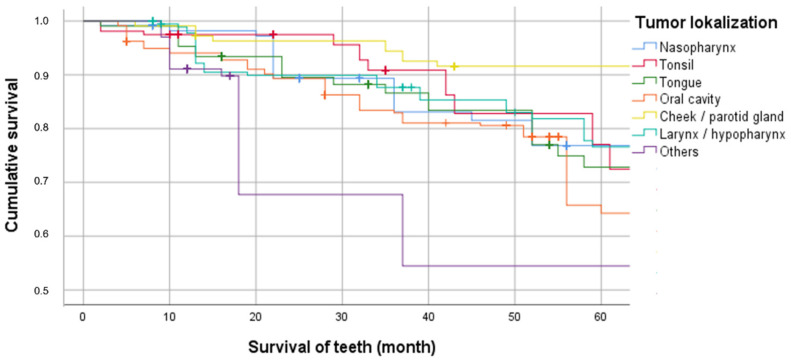
Cumulative survival of teeth in irradiated patients depending on the tumor localization.

Since tumor localization was taken as a surrogate for the location of the target volume or the highest irradiation dose, which often implies an increased irradiation dose to the areas of the jaws and tooth-bearing alveolar processes, the 5-YSP was calculated for all teeth of the irradiated patients in relation to tumor localization (Figure 2).

The teeth of patients with tumor localization “other”, e.g., CUP syndrome, had the lowest 5-YSP (54.5%; 95% CI: 42.7–66.3%), followed by oral cavity localization (65.8%; 95% CI: 58.4–73.2%). The teeth of patients irradiated for parotid or buccal mucosal malignancy achieved the highest 5-YSP (91.6%; 95% CI: 86.2–97.0%; *p* < 0.001).

The teeth of irradiated patients with “lowest SFR” (*n* = 461) exhibited a lower 5-YSP (63.9%; 95% CI: 58.9–68.9%) than teeth of patients with “regular” (*n* = 319, 82%; 95% CI: 77.0–87.0%) or “low SFR” (*n* = 270, 85.1%; 95% CI: 79.7–90.5%; *p* < 0.001). Mandibular teeth had a significantly lower 5-YSP (66.0%; 95% CI: 61.4–70.6%) than maxillary teeth (83.1%; 95% CI: 79.1–87.1%; *p* < 0.001). Teeth that were crowned after RT had significantly higher 5-YSP (94.3%; 95% CI: 90.7–97.9%) than those that were already crowned (74.3%; 95% CI: 66.5–82.1%) or not crowned (72.3%; 95% CI: 68.7–75.9%; *p* < 0.001).

Multivariable analysis of the survival of teeth of irradiated patients was performed using a marginal model. The analysis revealed a relevant influence on the survival of teeth of irradiated patients for different variables (Table 3). Age (3% increase in the risk of loss [RL] per year of life), SFR (55.4% reduction in the RL per mL of saliva), and RCT (2.2-fold increase in the RL) displayed a significant influence. Regular nicotine abuse and the presence of intraoral defects also increased the RL by 1.5 and 1.6 times, respectively. Teeth crowned after completion of RT exhibited a 77.1% lower RL compared with uncrowned teeth. The teeth of patients irradiated for nasopharyngeal carcinoma or, for example, CUP syndrome, showed a 1.7- and 2.0-fold higher RL, respectively, than teeth from patients irradiated for oral malignancy. Maxillary teeth displayed a 51.6% lower RL than mandibular teeth. Sex did not influence tooth survival after RT (Table 3).

#### 3.3.2. Factors Influencing the Survival of Teeth of Non-Irradiated Patients

The 5-YSP of the teeth of non-irradiated patients was also influenced by SFR. Due to the small number of teeth in the “low SFR” group (*n* = 12), these teeth were combined with those of the “lowest SFR” group (*n* = 83) and compared with the survival probabilities of the teeth of patients with regular SFR (*n* = 117). Here, the teeth of patients with regular SFR were found to have a higher 5-YSP (94.6%; 95% CI: 90.2–99.0%) than those of patients with low or lowest SFR (81.8%; 95% CI: 72.6–91.0%; *p* = 0.002). Maxillary teeth had a higher 5-YSP than mandibular teeth (94.5%; 95% CI: 89.5–99.5% vs. 84.3%; 95% CI: 76.9–91.7%, *p* = 0.779).

Multivariable analysis of the survival of teeth of non-irradiated patients was performed using a marginal model. The analysis showed an effect on survival only for the SFR (per mL additional saliva reduction in the RL of 77.3%). Since all dentate non-irradiated patients had an oral defect and had not received RCT, these variables were not included in the analysis (Table 4).

## 4. Discussion

In the present study, significant differences were identified in the survival probabilities of the teeth of irradiated and non-irradiated patients.

### 4.1. Extent of Extraction in Relation to TT

In both irradiated and non-irradiated patients, an average of four teeth had to be extracted related to TT. This is of interest because it is known that patients advised to have their teeth removed as part of the treatment preceding RT generally object and view it negatively [25]. Consequently, since the number of extracted teeth is comparable, it can be assumed that the need for rehabilitation is comparable in irradiated and non-irradiated patients or that there is a similar need for rehabilitation following TT. Therefore, the first part of the null hypothesis regarding comparable dental rehabilitation related to TT in irradiated and non-irradiated patients can be confirmed. However, relevant differences were observed regarding the long-term survival of teeth of irradiated and non-irradiated patients; hence, the second part of the null hypothesis must be rejected.

### 4.2. Factors Influencing the Survival Probability of Teeth after TT

Tooth structure defects were in most cases indicative of tooth removal. Despite close monitoring appointments, rapid progression of generalized (radiation) caries was observed in several patients. Generally, home oral hygiene is considered to play a key role in the prevention of caries. Particularly in cases of reduced salivary flow immediately after completion of RT, appropriate performance is usually difficult due to the vulnerable mucous membranes. Various toothpastes and rinsing solutions are currently available on the market, intending to support remineralization of the tooth hard substances [26]. Regular application of higher doses of fluoride to tooth surfaces via toothpaste or medication carriers has been a long-standing recommendation [27]. More recent approaches involve the use of casein phosphopeptides (CPP), which are derived from milk casein and can bind amorphous calcium phosphate (ACP) (CPP-ACP) [28,29]. However, no randomized prospective studies are currently available that demonstrate the efficacy of specific oral hygiene protocols or the use of specific toothpastes in high-risk patients. In the present study, although patients were regularly instructed and remotivated regarding adequate oral hygiene, there was no systematic review of the same. In this context, Jham et al. [30] reported that approximately 80% of patients did not follow the instructions of the dentist. Specifically for the group of oncological patients, it was highlighted that the relevance of dental instructions is often considered less important or not grasped cognitively in the face of life-threatening disease [3]. The lack of awareness of the need for meticulous oral hygiene could contribute significantly to caries progression. A study was conducted at Martin Luther University on possible factors influencing the occurrence of radiation caries, taking into account the level of education [6]. Here, it was found that patients with a lower level of education exhibited a higher risk of developing caries after TT. However, the level of education, compliance with regard to oral hygiene recommendations, and oral hygiene utensils used were not taken into account in the present study.

### 4.3. Radiation Therapy and Xerostomia

RT had a significant effect on the probability of tooth survival (74.2% 5-YSP of the teeth of irradiated patients vs. 89.4% of the teeth of non-irradiated patients). To our knowledge, there are no currently available studies in the literature that could be used to compare the results of this study.

The side effect profile of patients after RT is known to be significantly influenced by the localization of the tumor and the primary target volume. For example, if the lymph node levels IIa and IIb are included in the irradiation volume, a high dose impact on the parotid gland can be expected. With the implementation of IMRT and later VMAT, more targeted irradiation of the tumor tissue became possible. Hereby, in principle, improved sparing (ideally < 20 Gy) of the contralateral parotid gland can be achieved for all tumor localizations [31,32]. This has been shown to result in increased post-therapeutic salivary flow and a reduction in caries risk [5,31,32]. However, the results of the study illustrate that the extent of this reduction varies depending on the tumor sites. For example, teeth from patients who received higher doses of bilateral RT due to oral cavity carcinoma or CUP syndrome, by including the high cervical seated lymphatics on both sides of the neck in the RT volume, exhibited the lowest 5-YSP (54.5% vs. 65.8%). A considerably higher 5-YSP was observed for patients with malignancies in the parotid glands or buccal mucosa, in whom sparing of the parotid glands is usually possible (91.6%). This illustrates that tumor localization should be considered when assessing the prognosis of teeth of irradiated patients. Here, the tumor localization “oral cavity,” accounting for the largest percentage in the present study, could be considered a “high-risk group.” As a result of the cohort size and the present cluster effect due to the affiliation of different teeth to one patient, the tendencies shown in the Kaplan–Meier curves can only be evaluated critically. The cluster effect was taken into account later in the multivariable analysis.

The lowest SFR was present in 47.8% of the non-irradiated and 54.7% of the irradiated patients at the time of the examination. This indicates that even in patients with TT excluding RT, there are more patients who suffer from severe dry mouth with the associated negative consequences [2,33]. There were considerable dental survival differences in both irradiated and non-irradiated patients depending on the degree of oral dryness. The general relationship between reduced SFR and the occurrence of caries or tooth loss is also known from other medical conditions and their treatment-related side effects. For example, patients with Sjögren’s syndrome, in which a chronic autoimmune reaction leads to destruction of the salivary and lacrimal gland tissue, are considered to be at comparable risk of caries [34]. The association with the occurrence of dry mouth is also considered for certain groups of medications, e.g., antipsychotics, or the presence of diabetes [35,36,37]. Since a large proportion of the patients did not singularly suffer from a malignancy in the head and neck region, but also from various co-diseases treated by a wide variety of medications, dry mouth as a side-effect of co-medications is also quite plausible in the non-irradiated patients.

When interpreting the data, it must be taken into account that the development of dry mouth over time after TT cannot be reconstructed for the present study cohort. It is well known that the radiogenic decrease in salivary flow peaks at 6 months after irradiation, and by the end of the first year, most of the glandular recovery occurs [31,32]. Accordingly, xerostomia is a factor that can be estimated at the beginning of RT based on the tumor location and irradiation doses to the parotid glands, but its expression can only be assessed after one year. This time interval was considered in this study. Another limitation is due to the high inter- and intraindividual variations of the stimulated and unstimulated salivary flow independent of the measurement method. The amount of saliva recorded in the test is therefore usually a considerable scattering parameter, by which only tendencies can be detected.

### 4.4. Crowning

Teeth crowned after TT including RT had a considerably higher probability of survival than teeth crowned prior to TT or not crowned teeth (94.3% [95% CI: 90.7–97.9%] vs. 74.3% [95% CI: 66.5–82.1%] and 72.3% [95% CI: 68.7–75.9%]). Hereby, the survival probability of teeth crowned after TT found in this study is comparable to the data in the literature on the survival of teeth restored with single crowns whose wearers had not undergone TT [38,39]. Therefore, it can be concluded that favorable survival rates for crowned teeth are also achieved in irradiated patients if thoughtful treatment is implemented. However, it must be considered that teeth were only crowned after assessment of the “chronic side effect profile,” and if they were expected to have a reasonable prognosis one year after completion of TT. This indicates that critical teeth, which were more likely to be extracted in the group of uncrowned teeth, were not included in the group of teeth to be crowned after TT. Therefore, the conditions of the tooth groups can only be compared to a limited extent. The position of the preparation margin and its influence on the development of secondary caries could be responsible for the poorer survival probability of the teeth already crowned before TT. In particular, in crowned teeth with possibly discolored crown margins, secondary caries often spread unnoticed beneath the crown. The actual extent of the damage can often only be assessed after removal of the crown and, specifically during the fulminant phase of radiation caries, often means extraction of the affected tooth.

It must be highlighted that individual tooth types, vitality, or periodontal condition of the teeth before and after TT were not considered. Consequently, the results can only indicate the positive tendency of crowning after TT. Therefore, systematic investigation with consideration of time-dependent covariates should be the subject of future studies.

### 4.5. Difference between Upper and Lower Teeth

Mandibular teeth had a lower survival probability than maxillary teeth, especially in irradiated patients. In the normal population, an opposite trend can usually be observed. This is generally attributed to chronic periodontal disease, which often progresses more rapidly in the maxilla, and the protective effect of salivary flow, particularly in the region of the mandibular anterior teeth, due to the excretory ducts of the salivary glands. In patients with post-TT in the head and neck region, salivary flow is often reduced and is compounded by the fact that oral hygiene is often difficult, particularly in the mandible, due to anatomical changes and functional limitations. The mandibular region is also much more frequently integrated into the radiation therapy volume than the maxillary region due to the tumor localization. In combination with its monoarterial blood supply, the ORN risk for the mandible is generally considered to be much higher than that of the maxilla [12]. In the case of surgical ORN therapy, adjacent teeth are also more frequently removed in the course of plastic coverage of formerly necrotic bone sections.

### 4.6. Multivariable Analysis

Multivariable analyses performed separately for data on the survival time of the teeth of irradiated and non-irradiated patients only revealed the SFR as a common relevant factor influencing the RL. This decreased by 55.4% per mL of saliva for the teeth of non-irradiated patients and by 77.3% for the teeth of irradiated patients. In the case of teeth of irradiated patients, age, nicotine abuse, simultaneous chemotherapy, and tumor localization were additional factors increasing risk, whereas post-radiation crowning reduced the RL.

For age, an increased risk of 3% per year of life was determined. In older adults, a reduction in salivary flow with a preferentially high-carbohydrate diet has already been identified in the literature, in addition to a reduction in cognitive and manual skills, which complicate home oral hygiene and increase caries prevalence [9,40,41]. These factors may be more influential in patients after TT with RT as the oral mucosa remains more sensitive years after radiation and anatomic changes further complicate the accessibility to the teeth. This assumption is also supported by the regression results, as the RL in patients with intraoral defects was twice as high.

Sex showed no relevant influence on the risk of tooth loss in the present study, but smokers exhibited a 1.5-fold increased RL. Peterson and Twetman [10] also reported a 1.5-fold higher risk of tooth decay for smokers without a history of malignancy in their study. Therefore, our current findings are in agreement with the data in the literature. However, data on stimulant history should only be evaluated with reservations, as the information is primarily based on interviews with the inherent risk of participants providing socially desirable responses. In this respect, the apparent difference regarding nicotine abuse as a risk factor in the teeth of irradiated vs. non-irradiated patients must be put into perspective. The hazard ratio found for the factor “nicotine abuse” was in the 95% CI of the group of irradiated patients. Therefore, although the observed trend may not have been equally pronounced in both groups, a relevant opposite trend cannot be inferred.

An increase in the RL was further represented by the implementation of simultaneous chemotherapy. Chemotherapeutic agents are primarily applied intravenously and exert a systemic rather than a local effect by inhibiting DNA synthesis. In the oral region, the mucous membranes and salivary glands are particularly affected by chemotherapy [2]. However, these side effects tend to be acute in nature and usually subside after three months. According to current knowledge, there is still no causal relationship between simultaneous chemotherapy and an increased risk of tooth loss. However, in the present study, simultaneous chemotherapy was more likely to be applied to tumors of more advanced UICC grade, which means that the risk factor “chemotherapy” may possibly be considered as a surrogate factor for a more advanced tumor stage.

The final factor included in the analysis was tumor localization. In the analysis, the localization “oral cavity” was adjusted against, as this localization contained the most teeth and had already been found to exhibit high caries prevalence in a previous study [11]. Although in the univariable analysis, the teeth of patients with buccal mucosa or parotid carcinoma had the highest survival rates, this difference was no longer relevant when viewed multivariably. Here, only the tumor localizations “nasopharynx” and “other”, such as CUP syndromes, displayed an increased RL compared to the oral cavity. At this point, it must be emphasized that the number of teeth in the individual tumor localization groups differed, as already explained, and that only tendencies can be represented on the basis of the hazard ratios with comparatively robust 95% CIs. It can therefore be concluded that there are different RL depending on the tumor localization, but these must be evaluated and put into perspective in conjunction with the other risk factors. Prospective studies involving a much larger study cohort will be required to ascertain the influence of tumor localization on the risk of tooth loss.

In addition to the limitations already discussed, the study results are subjected to several biases. It should be considered that the underlying oncological disease of the cohort described in the study is associated with only a low survival probability of sometimes 60% or less after 5 years. Changes in dental status of a large proportion of patients who had undergone dental rehabilitation as part of tumor therapeutic head and neck irradiation could therefore not be considered. As the proportion of so-called long-term survivors might have been higher, the proportion of patients with long-term side effects as ORN might also have been higher than found in previous studies [12]. Since the data on the course of dental treatment were recorded retrospectively based on the patient records, the relevance of various influencing factors, such as diet, dental hygiene habits and the aids used, as well as the occurrence and medication of concomitant diseases, were not assessed. The evaluation of these additional factors would require prospective studies. In addition, no power analysis was performed. After recruitment of the maximum number of available patients, it was determined, supported by the Institute of Epidemiology, how many variables could be included in the multivariate analysis to achieve well-founded results with a comparatively small patient cohort. However, the results of this study will hopefully be the basis for the power analysis of further, at best multicenter and prospective studies, which will test and extend the conclusions presented in the present study.

## 5. Conclusions

Due to the relatively small number of patients, the comparatively large number of variables, and the varying observation periods, only tendencies can be shown. The following hypotheses can be generated based on the results of the present work:

The need for dental rehabilitation in patients undergoing TT in the head and neck region is comparable regardless of radiation therapy that may be administered in addition to or instead of surgical tumor resection. However, the probability of survival of the teeth decreases after the end of TT, particularly in irradiated patients. The most frequent reason for tooth extraction is advanced destruction of the dental hard tissues, mostly due to radiation caries. Advanced age, the presence of xerostomia, and defects in the oral cavity contribute to further reduction in the probability of tooth survival. In patients with these factors, if the prognosis of the remaining teeth is doubtful, preference should be given to implants.

## Figures and Tables

**Table 1:** Tumor localization and tumor stage according to ICD-10 and UICC.

Localization	ICD-10	UICC-Stadium							Number (%)
		I	II	III	IV A	IV B	IV C	n.d.	
Nasopharynx	C11, C30, and C31	0	1	0	2	2	0	2	7 (5.9)
Tonsil	C09 and C10	0	2	1	6	4	0	1	14 (11.9)
Tongue base	C01	0	2	1	4	0	0	1	8 (6.8)
Oral cavity	C00, C02-C06, and C08	9	13	11	12	6	1	5	57 (48.3)
Cheek/parotid gland	C06 and C07	3	1	3	1	0	0	0	8 (6.8)
Larynx/hypopharynx	C12, C13, and C32	0	5	4	7	0	0	0	16 (13.6)
Others	C41, C49, C80, and D18	1	0	0	2	2	0	3	8 (6.8)
Number (%)		13 (11.0)	23 (19.5)	21 (17.8)	34 (28.8)	14 (11.9)	1 (0.8)	12 (10.2)	118 (100)

ICD, International Statistical Classification of Diseases and Related Health Problems; UICC, International Union Against Cancer; n.d., not defined.

**Table 2:** Scope of dental treatment in dentate patients in relation to TT, with and without RT.

Type of TT	Number of Patients	Number of Teeth	Removed Teeth			
			∑	Mean	Min.	Max.
TT without RT	Dentate patients (prior to dental treatment, *n* = 20; after dental treatment, *n* = 18)	300	94	4.09	0	21
	Edentulous patients (prior to dental treatment, *n* = 3; after dental treatment, *n* = 5)	0				
TT with RT/RCT	Dentate patients (prior to dental treatment, *n* = 79; after dental treatment, *n* = 69)	1406	384	4.04	0	23
	Edentulous patients (prior to dental treatment, *n* = 16; after dental treatment, *n* = 26)	0				

TT, tumor therapy; RT, radiotherapy; RCT, radiochemotherapy.

**Table 3:** Hazard ratios for demographic and clinical characteristics of irradiated patients for survival of teeth.

Variables	Reference	Hazard Ratio	95% CI		*p*-Value
Age	-	1.030	1.011	1.049	0.002
Sex	Male	0.734	0.305	1.776	0.494
Nicotine abuse	No nicotine abuse	1.505	0.769	2.946	0.233
RCT	No CT	2.237	1.314	3.806	0.003
SFR	-	0.446	0.220	0.905	0.025
Assignment to jaw	Lower jaw	0.484	0.315	0.743	0.001
Intraoral defects	No intraoral defects	1.573	0.817	3.027	0.175
Crowning state					
Crowning prior to RT	No crowning	1.890	0.977	3.657	0.059
Crowning after RT		0.229	0.088	0.599	0.003
Tumor localization					
Tonsil	Oral cavity	1.197	0.575	2.492	0.631
Togue base		1.129	0.456	2.794	0.794
Nasopharynx		1.735	0.380	7.923	0.477
Parotid gland/cheek		0.636	0.259	1.572	0.328
Larynx		1.433	0.593	3.461	0.424
Others		2.070	0.697	6.147	0.190

CT, chemotherapy; RCT, combined radiotherapy and chemotherapy; RT, radiotherapy; SFR, salivary flow rate; CI, confidence interval.

**Table 4:** Hazard ratios of demographic and clinical characteristics of non-irradiated patients for survival of teeth.

Variables	Reference	Hazard Ratio	95% CI		*p*-Value
Age	-	1.010	0.929	1.100	0.809
Sex	Male	1.096	0.089	13.417	0.943
Nicotine abuse	No nicotine abuse	0.544	0.048	6.212	0.624
SFR	-	0.227	0.051	1.010	0.052
Assignment to jaw	Lower jaw	0.972	0.116	8.122	0.979
Crowning state					
No crowning	Crowning prior to RT	1.933	0.147	25.461	0.616
Crowning after RT		1.199	0.388	3.699	0.753

SFR, salivary flow rate; RT, radiotherapy; CI, confidence interval.

## Data Availability

The data presented in this study are available on request from the corresponding author due to privacy restrictions.

## References

[1] Marker P., Siemssen S.J., Bastholt L. (1997). Osseointegrated implants for prosthetic rehabilitation after treatment of cancer of the oral cavity. Acta Oncol..

[2] Epstein J.B., Thariat J., Bensadoun R.J., Barasch A., Murphy B.A., Kolnick L., Popplewell L., Maghami E. (2012). Oral complications of cancer and cancer therapy: From cancer treatment to survivorship. CA Cancer J. Clin..

[3] Abed H., Reilly D., Burke M., Daly B. (2019). Patients with head and neck cancers’ oral health knowledge, oral health-related quality of life, oral health status, and adherence to advice on discharge to primary dental care: A prospective observational study. Spec. Care Dent..

[4] Schweyen R., Hey J., Fränzel W., Vordermark D., Hildebrandt G., Kuhnt T. (2012). Radiation related caries: Etiology and possible preventive strategies. What should the radiotherapist know?. Strahlenther Onkol..

[5] Hey J., Seidel J., Schweyen R., Paelecke-Habermann Y., Vordermark D., Gernhardt C.R., Kuhnt T. (2013). The influence of parotid gland sparing on radiation damages of dental hard tissues. Clin. Oral Investig..

[6] Schweyen R., Hey J., Seidel J., Wienke A., Paelecke-Habermann Y., Vordermark D., Kuhnt T. (2015). Impact of parotid gland dose and patient-related factors on radiation damage to dental hard tissues. J. Dent. Sci..

[7] Jansma J., Vissink A., Spijkervet F.K., Roodenburg J.L., Panders A.K., Vermey A., Szabo B.G., Gravenmade E.J. (1992). Protocol for the prevention and treatment of oral sequelae resulting from head and neck radiation therapy. Cancer.

[8] Selwitz R.H., Ismail A.I., Pitts N.B. (2007). Dental caries. Lancet.

[9] Muñoz-González C., Vandenberghe-Descamps M., Feron G., Canon F., Labouré H., Sulmont- Rossé C. (2018). Association between salivary hypofunction and food consumption in the elderlies. A systematic literature review. J. Nutr. Health Aging.

[10] Petersson G.H., Twetman S. (2019). Tobacco use and caries increment in young adults: A prospective observational study. BMC Res. Notes.

[11] Schweyen R., Stang A., Wienke A., Eckert A., Kuhnt T., Hey J. (2017). The influence of dental treatment on the development of osteoradionecrosis after radiotherapy by modern irradiation techniques. Clin. Oral Investig..

[12] Kuhnt T., Stang A., Wienke A., Vordermark D., Schweyen R., Hey J. (2016). Potential risk factors for jaw osteoradionecrosis after radiotherapy for head and neck cancer. Radiat. Oncol..

[13] Grötz K.A. (2002). Zahnärztliche Betreuung von Patienten mit tumortherapeutischer Kopf-Hals- Bestrahlung. Die Deutsche Zahnärztliche Zeitschrift.

[14] Sulaiman F., Huryn J.M., Zlotolow I.M. (2003). Dental extractions in the irradiated head and neck patient: A retrospective analysis of Memorial Sloan-Kettering Cancer Center protocols, criteria, and end results. J. Oral. Maxillofac. Surg..

[15] Witter D.J., van Palenstein Helderman W.H., Creugers N.H., Käyser A.F. (1999). The shortened dental arch concept and its implications for oral health care. Community Dent. Oral Epidemiol..

[16] Epstein J.B., Chin E.A., Jacobson J.J., Rishiraj B., Le N. (1998). The relationships among fluoride, cariogenic oral flora, and salivary flow rate during radiation therapy. Oral Surg. Oral Med. Oral Pathol. Oral Radiol. Endod..

[17] Bacher H., Schweyen R., Kuhnt T., Leplow B., Hey J. (2020). Use of a patient information leaflet on oro-dental care during radiotherapy. Patient Prefer Adher..

[18] Bacher H., Schweyen R., Vordermark D., Leplow B., Hey J. (2020). Development and validation of an information leaflet on oral care for irradiated patients. Patient Prefer Adher..

[19] Kielbassa A.M., Hinkelbein W., Hellwig E., Meyer-Luckel H. (2006). Radiation-related damage to dentition. Lancet Oncol..

[20] Grötz K.A., Riesenbeck D., Brahm R., Seegenschmiedt M.H., al-Nawas B., Dörr W., Kutzner J., Willich N., Thelen M., Wagner W. (2001). Chronic radiation effects on dental hard tissue (radiation caries). Classification and therapeutic strategies. Strahlenther Onkol..

[21] Nicoletti G., Soutar D.S., Jackson M.S., Wrench A.A., Robertson G. (2004). Chewing and swallowing after surgical treatment for oral cancer: Functional evaluation in 196 selected cases. Plast. Reconstr. Surg..

[22] Burlage F.R., Coppes R.P., Meertens H., Stokman M.A., Vissink A. (2001). Parotid and submandibular/sublingual salivary flow during high dose radiotherapy. Radiother. Oncol..

[23] Dawes C. (1987). Physiological factors affecting salivary flow rate, oral sugar clearance, and the sensation of dry mouth in man. J. Dent. Res..

[24] Wei L.J., Lin D.J., Weissfeld L. (1989). Regression analysis of multivariate incomplete failure time data by modeling marginal distributions. J. Am. Stat. Assoc..

[25] Parahoo R.S., Semple C.J., Killough S., McCaughan E. (2019). The experience among patients with multiple dental loss as a consequence of treatment for head and neck cancer: A qualitative study. J. Dent..

[26] Su N., Marek C.L., Ching V., Grushka M. (2011). Caries prevention for patients with dry mouth. J. Can. Dent. Assoc..

[27] Beech N., Robinson S., Porceddu S., Batsone M. (2014). Dental management of patients irradiated for head and neck cancer. Aust. Dent. J..

[28] Farooq I., Moheet I.A., Imran Z., Farooq U. (2013). A review of novel dental caries preventive material: Casein phosphopeptide–amorphous calcium phosphate (CPP–ACP) complex. King Saud Univ. J. Dent. Sci..

[29] Guggenheim B., Schmid R., Aeschlimann J.M., Berrocal R., Neeser J.R. (1999). Powdered milk micellar casein prevents oral colonization by *Streptococcus sobrinus* and dental caries in rats: A basis for the caries-protective effect of dairy products. Caries Res..

[30] Jham B.C., Reis P.M., Miranda E.L., Lopes R.C., Carvalho A.L., Scheper M.A., Freire A.R. (2008). Oral health status of 207 head and neck cancer patients before, during and after radiotherapy. Clin. Oral Investig..

[31] Hey J., Setz J., Gerlach R., Janich M., Sehlleier S., Schaller H.G., Gernhardt C.R., Kuhnt T. (2009). Parotid-gland-sparing 3D conformal radiotherapy in patients with bilateral radiotherapy of the head and neck region—Results in clinical practice. Oral Oncol..

[32] Hey J., Setz J., Gerlach R., Janich M., Hildebrandt G., Vordermark D., Gernhardt C.R., Kuhnt T. (2011). Parotid gland-recovery after radiotherapy in the head and neck region—36 months follow-up of a prospective clinical study. Radiat. Oncol..

[33] Deng J., Jackson L., Epstein J.B., Migliorati C.A., Murphy B.A. (2015). Dental demineralization and caries in patients with head and neck cancer. Oral Oncol..

[34] Zero D.T., Brennan M.T., Daniels T.E., Papas A., Stewart C., Pinto A., Al-Hashimi I., Navazesh M., Rhodus N., Sciubba J. (2016). Clinical practice guidelines for oral management of Sjögren disease: Dental caries prevention. J. Am. Dent. Assoc..

[35] Hu K.F., Chou Y.H., Wen Y.H., Hsieh K.P., Tsai J.H., Yang P., Yang Y.-H., Lin C.-H.R. (2016). Antipsychotic medications and dental caries in newly diagnosed schizophrenia: A nationwide cohort study. Psychiatry Res..

[36] Mauri-Obradors E., Estrugo-Devesa A., Jané-Salas E., Viñas M., López-López J. (2017). Oral manifestations of diabetes mellitus. A systematic review. Med. Oral Patol. Oral Cir. Bucal..

[37] Quilici D., Zech K.N. (2019). Prevention and treatment options for medication-induced xerostomia. Gen. Dent..

[38] Sailer I., Makarov N.A., Thoma D.S., Zwahlen M., Pjetursson B.E. (2015). All-ceramic or metalceramic tooth-supported fixed dental prostheses (FDPs)? A systematic review of the survival and complication rates. Part I: Single crowns (SCs). Dent. Mater..

[39] Suksaphar W., Banomyong D., Jirathanyanatt T., Ngoenwiwatkul Y. (2018). Survival rates from fracture of endodontically treated premolars restored with full-coverage crowns or direct resin composite restorations: A retrospective study. J. Endodont..

[40] Gupta A., Epstein J.B., Sroussi H. (2006). Hyposalivation in elderly patients. J. Can. Dent. Assoc..

[41] Chávez E.M., Wong L.M., Subar P., Young D.A., Wong A. (2018). Dental care for geriatric and special needs populations. Dent. Clin. N. Am..

